# Chronic Eosinophilic Pneumonia with Cardiac Involvement

**Published:** 2018-02

**Authors:** Fariba Mansouri, Sina Moradmand, Amirsobh Rakhshankhah

**Affiliations:** 1 Department of Respiratory, Amir Alam Hospital, Tehran University of Medical Sciences, Tehran, Iran; 2 Department of Cardiology, Amir Alam Hospital, Tehran University of Medical Sciences, Tehran, Iran

**Keywords:** Eosinophilic pneumonia, Dyspnea, Cardiac involvement

## Abstract

Chronic Eosinophilic Pneumonia (CEP) is an idiopathic disorder characterized by an abnormal marked accumulation of eosinophils in the interstitial and alveolar spaces of the lung. CEP is typically suspected in a patient with progressive dyspnoea over one to four months and a chest radiograph showing bilateral peripheral or pleural-based opacities. Dominant extrapulmonary manifestations in CEP are rare. We report a 44-year-old Iranian woman with progressive dyspnea, peripheral chest opacity, and cardiac involvement. A diagnosis of CPE was considered base on clinical and para clinical criteria.

## INTRODUCTION

Chronic Eosinophilic Pneumonia (CEP) is an interstitial lung disease in which eosinophils are the most prominent inflammatory cells on histopathology examination. Other inflammatory cells, especially lymphocytes and neutrophils, are often present but eosinophils are clearly predominant (1). CEP mostly occurs in women with a mean age of 45 years at diagnosis. A previous history of asthma is present in up to two thirds of the patients. The most common respiratory symptoms are cough, dyspnea, and chest pain. Although CEP is not a systemic disease, isolated and usually moderately severe extra pulmonary manifestations have been occasionally reported (2). We report a 44-year-old Iranian woman with a diagnosis of CEP and cardiac involvement. After ruling out other known causes, diagnosis of CEP was made.

## CASE SUMMARY

**Figure 1.**
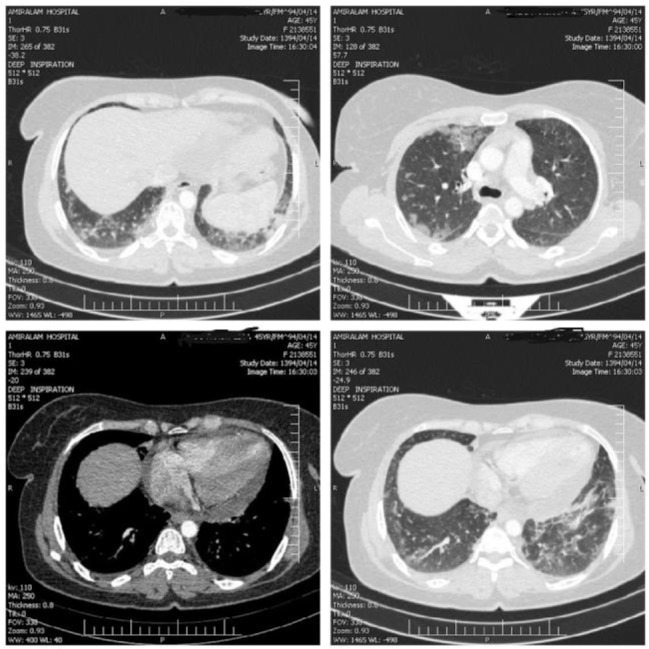
Chest spiral CT scan image of patient.

A 44-year-old Iranian woman was admitted to Amiralam Educational Hospital complaining of dyspnea that had begun two months prior to admission. She noticed dry coughs, chest tightness, and pain in the left subscapular region, but she had no fever, chills, hemoptysis, nausea, vomiting, diaphoresis, night sweats, weight loss, anorexia, joint pain, or rashes. Dyspnoea gradually increased in severity. The patient had asthma and mild allergic rhinitis for more than two years and regularly visited an asthma clinic. She was treated with fluticasone/salmeterol propionate inhaler and fluticasone nasal spray. She had her usual activities until two months before admission. The family history of the patient was negative. On initial examination, the vital signs were normal and a generalized wheeze was heard in both lungs. There was no tenderness in the left scapula or arm on palpation. The remainder of the examination was normal. Oxygen saturation was 93% in room air. A chest radiograph revealed cardiomegaly and peripheral opacities in both lungs without pleural effusion. Computed tomography of the chest showed multiple patchy ground-glass opacities and consolidations in both lungs, predominantly in the peripheral and upper lung zone, cardiomegaly, mild pericardial thickening, and pericardial effusion. The central airway was clear and there was no evidence of a pulmonary embolus, hypertension, and lymphadenopathy (Figure 1). Electrocardiograms taken on admission and two days later revealed T inversion on precordial and lateral leads with a normal sinus rhythm.

Complete blood count and differential blood test confirmed hypereosionphilia. Hematologic investigations showed a high level of C reactive protein and erythrocyte sedimentation rate (Table 1). Renal function tests, urinalysis, and electrolytes were all within the normal limit.

**Table 1.** Laboratory data

**Variable**	**Value**	**Reference range**
White blood cell count(per mm^3^)	13/6×10^3^	4–10×10^3^
Hemoglobin (g/dL)	10/3	12–16
Hematocrit (%)	32	37–52
Platelet count(permm^3^)	574	150–400×10^3^
Neutrophil (%)	28	50–65
Lymphocyte (%)	21	10–25
Monocyte (%)	2	2–10
Eosinophil (%)	49	2–5
Urea nitrogen (mg/dL)	16	10–45
Creatinine (mg/dL)	0.8	0.7–1/4
Alanine aminotransferase (U/lit)	20	5–40
Aspartate aminotransferase (U/lit)	28	5–40
Lactic dehydrogenase (U/lit)	836	225–500
Troponin I (ng/mL)	0.8	Up to o.1
Creatine Kinase MB isoenzyme (ng/mL)	34.8	<24
Erythrocyte sedimentation rate (mm/h)	64	<25
C reactive protein (mg/lit)	86	<10
Alkaline phosphatase (U/lit)	265	64–360

Antinuclear Antibodies (ANA) were weakly positive. Anti-double stranded DNA, antineutrophilic cytoplasmic antibodies and other collagen vascular markers were negative at presentation and 3 and 6 months later. HIV antibody was negative and urine examination was unremarkable for illicit drugs. Cardiac troponin, creatine kinase MB, and Lactic Dehydrogenase (LDH) levels were high (Table 1).

**Figure 2.**
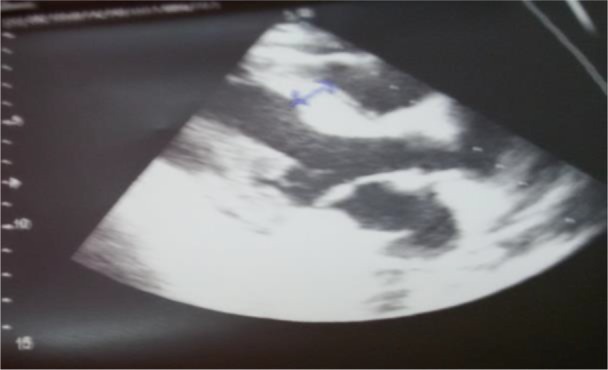
Echocardiography image of patient.

An echocardiogram was done one day after admission which revealed mild left diastolic and systolic dysfunction, increased septal and pericardial thickness, and mild pericardial effusion. The left ventricular ejection fraction was 45% (Figure 2).

TB skin test and laboratory parasitological assessments were negative. Abdominal ultrasound was normal without organomegaly and lymphadenopathy. There was no evidence of fungal, bacterial, or viral infection in the sputum samples and blood tests. Because of cardiac involvement, fiberoptic bronchoscopy was not allowed by the cardiologist.

Based on the clinical symptoms, a medical history of asthma and allergic rhinitis combined with characteristic CT findings, blood eosinophilia, sputum eosinophilia, and no evidence of any infection, collagen vascular diseases or malignancy, the patient was diagnosed with CEP.

Due to cardiac involvement, we decided to treat the patient with high dose prednisolone. The patient was started on methyl prednisolone 1 gr daily for three consecutive days, followed by prednisolone 1 mg/kg/day. Radiologic, echocardiographic, electrocardiogram and laboratory findings became normal after one month. Prednisolone was tapered gradually over six months; then, the patient was treated with fluticasone/salmeterol combination for asthma. During 24 months follow-up after cessation of systemic corticosteroids, the patient was stable without exacerbation of asthma or relapse of radiologic and laboratory findings.

## DISCUSSION

CEP is typically suspected in a patient with progressive dyspnoea over one to four months and a chest radiograph showing bilateral peripheral or pleural-based opacities. Among the first steps in the evaluation is to inquire whether the patient is taking or has recently taken any of the drugs associated with pulmonary eosinophilia or has resided in an area with an increased likelihood of exposure to endemic parasites or fungi (3).

A diagnosis of CEP is typically made based on a combination of clinical presentations, chest imaging showing predominantly peripheral or pleural-based opacities in the mid to upper lung zone, and a Bronchoalveolar Lavage (BAL) showing eosinophilia (≥25%). Lung biopsy is not necessary unless the BAL does not show eosinophilia, radiographic features are atypical, or the patient does not respond promptly to systemic glucocorticoid therapy (4).

The differential diagnoses of CEP include eosinophilic lung diseases of other etiologies and non eosinophilic lung diseases with similar clinical presentations.

Acute eosinophilic pneumonia differs from CEP in its acute, often fulminant onset (one month or less), severity of hypoxemia, typical absence of peripheral blood eosinophilia, and a diffuse pattern of radiographic opacities (5).

Allergic Bronchopulmonary Aspergillosis (ABPA) is similar to CEP in the combination of asthma, peripheral blood eosinophilia, and radiographic abnormalities in the upper lung zone. However, the radiographic opacities in ABPA are more typically those of bronchiectasis (“tram-tracking” or more centrally located mucus plugging “finger-in-glove”) (6). The diagnosis of ABPA is based on a serum IgE level >1000 IU/L, positive IgG specific to Aspergillus, and a positive skin prick test to Aspergillus antigen (5,6)

Eosinophilic granulomatosis with polyangiitis (EGPA, Churg-Strauss) is a vasculitic disorder characterized by sinusitis, asthma, and prominent peripheral blood eosinophilia. EGPA can have a similar presentation to CEP, although the radiographic opacities are typically seen in the mid rather than upper zone and are centre lobular rather than peripheral. EGPA is more likely to have extra pulmonary manifestations (e.g. skin, heart, and kidney). However, an overlap between CEP and EGPA has been noted, suggesting that CEP may be a presenting feature of EGPA (7).

No prospective trials have evaluated prednisone regimens in patients with CEP. An adequate initial therapy for virtually all patients with CEP consists of oral prednisone at a dose of 0.5 mg/kg/day (8).

For patients with a rapidly progressive disease (especially if associated with respiratory failure), high dose glucocorticoid therapy for 3–5 days, such as methylprednisolone 250 mg every six hours administered intravenously, has been recommended prior to switching to oral therapy (9).

Clinical improvement is often dramatic and rapid in response to prednisone, with profound symptomatic relief occurring in many patients within 48 hours. Patients with CEP are uniformly responsive to intravenous or oral glucocorticoids. Thus, an alternative diagnosis should be entertained if a patient does not improve upon receiving glucocorticoid treatment (5).

Although CEP is not a systemic disease, isolated and moderately sever extrapulmonary manifestations, such as pericarditis and repolarization (ST-T) abnormalities on an EEG, have been reported (3). In the present case, T inversion on precordial and lateral leads and pericardial thickening were seen.

Extrapulmonary manifestations suggest an overlap to CEP and eosinophilic granulomatosis with polyangiitis or Churg-Strauss diseases, eosinophilic pneumonia similar to CEP may be a presenting feature of EGPA. However, in this case, serologic markers for EGPA on admission and after 3 and 6 months were negative, and no other manifestations of vasculitis were noted. Moreover, after discontinuation of oral corticosteroid therapy, the patient was followed for 18 months with no systemic medication, and no clinical, laboratory, or radiologic problems compatible with EGPA or another diagnosis were detected.

Based on the clinical scenario provided, our unifying diagnosis for this patient was CEP. The keys to this diagnosis were a typical history and radiological signs, blood and sputum eosinophilia, ruling out of other diagnoses, and finally a very good response to treatment.
